# Effect of Tailored Coaching on Physicians’ Electronic Health Record Proficiency and User Experience: A Randomized Crossover Study

**DOI:** 10.1016/j.mcpdig.2023.02.005

**Published:** 2023-04-06

**Authors:** Saif Khairat, Prabal Chourasia, Elizabeth Kwong, Ji Min Choi, Carl Seashore

**Affiliations:** a Carolina Health Informatics Program, University of North Carolina at Chapel Hill, Chapel Hill, NC; b School of Nursing, University of North Carolina at Chapel Hill, Chapel Hill, NC; c Department of Statistics and Operations Research, University of North Carolina at Chapel Hill, Chapel Hill, NC; d Division of General Pediatrics and Adolescent Medicine, University of North Carolina at Chapel Hill, Chapel Hill, NC

## Abstract

**Objective:**

To examine the effect of a tailored EHR coaching intervention, informed by audit log data, on physicians’ EHR proficiency, and user experience among pediatricians.

**Patients and Methods:**

A 12-month (August 2020 – August 2021) randomized crossover quality improvement study of a tailored EHR coaching was conducted on 34 pediatric physicians at a major medical center. Participants were randomized into Group AB or Group BA. The intervention was a single 1-hour, one-on-one coaching session. An Epic certified pediatrician tailored each coaching session to meet each physician needs using their EHR audit log data. We analyzed EHR audit log data for 3 months pre- and post-intervention.

**Results:**

Out of the 34 physicians, 15 (44%) were primary care pediatricians, 19 (56%) were female, 24 (70%) practiced at the medical center. During the initial intervention, the average proficiency score for the Group AB increased by 8.9% (pre-post difference: 0.37, 95% CI: −0.35 to 1.09; *P*-value=.381). For the crossover intervention, the average proficiency score for Group BA significantly increased by 11.2% (pre-post difference: 0.31, 95% CI: −0.11 to 0.74; *P*-value=.05). The average perceived EHR workload decreased post coaching sessions compared to pre-session (50.89 vs 46.66, *P*-value=.06). Post-coaching intervention, the average score for perceived EHR usability improved compared to pre-intervention (4.10 vs 4.34; *P*-value=.1).

**Conclusion:**

Electronic health record audit log data can be used to inform tailored coaching to improve physicians’ EHR proficiency levels and user experience. Tailored EHR coaching can increase awareness about efficiency tools available in the EHR. These findings are relevant to decisionmakers and learning health systems interested in provider well-being by optimizing EHR use.

Approximately 40% of physicians report frustration with current electronic health record (EHR) interfaces and usage.^1^, ^2^, ^3^ Physicians experience physiologic fatigue because of inefficient use of EHRs (ie, spending too much time on EHRs).^4^^,^^5^ Inefficient use of EHRs is caused, in part, by current ineffective, generic EHR training mechanisms^3^ and result in reduced physician satisfaction, increased burnout, and decreased quality of care.^3^^,^^6^, ^7^, ^8^ Electronic health record training is a critical part of physician onboarding before the initiation of patient care in EHR-based clinics and hospitals.^9^^,^^10^ Improving EHR skills with training has been shown to reduce physicians’ daily workload and time interacting with EHRs in addition to improving their satisfaction and well-being.^6^^,^^7^^,^^11^, ^12^, ^13^

The current EHR training methods for physicians include classroom training (CRT),^14^ computer-based training (ie, simulation-based training),^15^ and blended training (mix of CRT and computer-based training methods),^16^ with CRT being the most used and least effective training method.^16^^,^^17^ One-on-one EHR training has the highest satisfaction and perceived effectiveness in improving EHR use.^16^^,^^17^ However, current EHR coaching programs use self-reported data (eg, surveys) to tailor EHR training interventions.^16^^,^^18^ In addition, training methods over time have shifted away from classroom-based training for EHR education between 2010 and 2020^19^; however, objective assessments of individual physician needs can further enhance EHR training.^20^

Studies have measured physician satisfaction using self-reported surveys^11^ to measure competency gaps using online need assessment^21^ and satisfaction surveys to assess the training outcomes of an EHR efficiency workshop.^22^ Although these studies demonstrated the use of subjective measures, the use of objective measures may reduce certain biases associated with self-reported surveys, such as reporting bias, selection bias, and recall bias.^23^ Studies using objective methods to tailor EHR training interventions may provide more reliable and reproducible results.^24^^,^^25^

Electronic health record audit log datasets capture and time stamp user activity when logged into these EHRs and offer user-centric insights into EHR usage.^23^^,^^26^ For instance, audit logs can be used to determine users’ level of proficiency and assess EHR usage by assessing where physicians spend most of their time in EHRs.^26^, ^27^, ^28^ Epic provides a summary of individual physician EHR audit log data through a tool called Signal.^29^ Signal provides a proficiency score for each provider, which is a vendor-generated monthly score based on how frequently a provider uses Epic efficiency tools (eg, customized speed buttons, chart search, SmartTools to compose notes, and preference lists).

Previous studies have used EHR audit logs to observe a wide range of clinical activities; however, these studies assessed general EHR use, clinical workflows extending beyond EHRs, and care team dynamics.^30^, ^31^, ^32^, ^33^ To date, systematic investigation of EHR audit log usage to tailor EHR coaching has not been assessed.

The objective of our study was to examine the effect of tailored coaching, informed by audit logs, on physicians’ EHR proficiency as well as user experience among primary care and specialty pediatricians.

## Methods

### Study Design

We conducted a 12-month randomized crossover trial (AB-BA design) testing the effect of tailored coaching on pediatric physicians’ EHR proficiency levels and user experience using a leading EHR system (Epic; Epic Systems). The crossover design was selected to further examine the effect of tailored coaching on physicians and increase statistical power. Coaching sessions were conducted between November 10, 2020, and April 27, 2021. We compared 3-month Epic Signal data, EHR audit log, and data before and after the coaching sessions to assess the effectiveness of the intervention. The study team included 2 clinical informaticians (C.S., S.K.) and 4 research assistants. The EHR coach (C.S.) was an Epic-certified physician builder and practicing pediatrician at the University of North Carolina at Chapel Hill. Approval from the University of North Carolina Institutional Review Board was obtained. This study followed the Standards for Quality Improvement Reporting Excellence reporting guidelines.^34^

### Participants and Settings

The study was conducted at a Southeastern US tertiary academic medical center. We recruited primary care and specialty pediatricians through departmental emails and flyers during October 2020. The eligibility criteria were as follows: (1) full-time employment in the pediatrics department (ie, faculty) and (2) prior experience using the institutional EHR (Epic) system. The participants were compensated for their participation.

We recruited a total of 34 pediatricians for this study. Our sample size falls within the range of previous coaching studies.^11^^,^^21^^,^^22^ After providing written informed consent, the participants were randomized in a ratio of 1:1 to either an intervention group (group AB) that underwent a 6-month intervention and a 1- to 2-week washout period, followed by a 6-month control period, or a control group (group BA) that underwent a 6-month control period and a 1- to 2-week washout period, followed by a 6-month intervention period.

### Intervention Design

After recruitment, we obtained 3 months of Epic Signal data for both groups AB (intervention) and BA (control) from August to October 2020. Then, we conducted 1-hour tailored coaching sessions for group AB as the initial intervention phase in November 2020. Afterward, we obtained 3 months of Signal data for both groups AB and BA from December 2020 to February 2021.

Before the coaching sessions, the participants completed 2 baseline surveys for the measurement of user experience: perceived workload (NASA Task Load Index [NASA-TLX]) and usability (Usability, Satisfaction, and Ease of Use [USE]).

In November 2020, each participant in group AB received a single 1-hour coaching session intervention. For each session, the Epic coach (C.S.) reviewed Signal for evidence of inefficiency, work outside of work hours, note length, smart tool, and the use of other customization. The coach tailored each session to focus on documentation burden or time-based measures. For each participant, we obtained 7 Signal metrics, of which 2 were time-based (ie, time in system, time in notes, time in basket, time in chart review, and pajama time) and 2 were documentation-based metrics (ie, note length and documentation length). All the Signal metrics were adjusted by appointment to account for differences in workload among the participants. For each participant, the coach identified the area of improvement, either time based or documentation based, and then tailored the session based on the identified EHR need.

The coach started each session with the participants self-identifying areas of frustration or perceived weakness and then shared Signal data, noting whether their self-perception and the Signal data aligned or identified different opportunities. During the coaching session, the audit log data were shared, explained, and reviewed in the context of the participants’ clinical work environments. Each participant was also asked to share their own perceived struggles (“pebbles in their shoes”) with EHR use. The participant and coach chose 2-4 topics to explore during the session and made real-time changes in their EHR profile (eg, improving templates and search filters). The coaching session was then tailored to include each participant’s perceived struggles in conjunction with their audit log data to create 2-3 actionable changes they could adopt with a goal toward improved efficiency with EHR use. The changes included more efficient data gathering, note composition, in-basket management, and staff communication. After each session, the coach sent a follow-up email with the topics covered along with the participant’s Signal data. Because of the coronavirus disease 2019 pandemic, these sessions were conducted over Zoom with screen sharing and recorded for later review if needed.

After the coaching sessions, we obtained 3 months of Epic Signal data for the participants. Then, we compared preintervention and postintervention Signal data for groups AB and BA to determine the effect of tailored coaching on the desired outcomes. The participants completed the follow-up surveys (TLX and USE) 3 months after receiving the intervention, which were used to compare changes in user experience before and after the intervention.

Next, in the crossover intervention phase, we switched the group assignment such that group AB became the control group and group BA became the intervention group. The crossover phase occurred between February and August 2021. The coaching intervention was delivered to group BA participants in May 2021 using the same procedures as the initial intervention phase.

### Outcomes

The primary outcomes were EHR proficiency level, measured using the proficiency score from Signal data, and user experience, measured using NASA-TLX (EHR workload) and USE (EHR usability).

### Materials and Data Sources

We obtained EHR proficiency scores from Epic Signal data for each participant for 3 months before and 3 months after the initial and crossover interventions.

We used 2 validated surveys to assess the effectiveness of the coaching program on physicians’ workload and usability. We administered a baseline survey after recruitment of the participants and an exit survey 3 months after the coaching session. We used the NASA-TLX^35^ and USE^36^ surveys in this study. The NASA-TLX survey consists of 6 categories of questions related to “mental demand,” “physical demand,” “temporal demand,” “performance,” “effort,” and “frustration.” Each category is rated on a 20-point scale, and the overall workload score ranges from 0 to 100. The NASA-TLX survey was used to assess physicians’ perceived EHR workload. A low NASA-TLX score indicates lesser workload.

The USE survey has 4 categories of measures about Epic usability: “use,” “ease of use,” “ease of learning,” and “satisfaction.” Each of these categories is rated on a 10-point Likert scale, and the overall usability score ranges from 0 to 10. The USE survey was designed to assess physicians’ perceived EHR usability, wherein a higher score indicates higher satisfaction.

### Analysis

We conducted a descriptive analysis to further analyze the survey and audit log data results between the pretailored and posttailored coaching sessions. We calculated the average proficiency score for the 3 months before the participants received the coaching intervention. Then, we computed the average proficiency score for the 3 months after they received the coaching intervention. We calculated the difference and percent change between the preintervention and postintervention proficiency scores. We removed missing values while computing the average, eg, if there was a missing value within the 3 months, we computed the average for 2 months. To compare the 2 independent samples, we applied the Wilcox Rank test instead of the paired *t* test to assess whether the group mean ranks differed between before and after the coaching intervention, taking into account our relatively smaller sample size of 34 physicians.^37^

The surveys were administered only to the intervention groups to measure changes in the physicians’ perceptions of EHRs before and after the intervention; therefore, our analysis included responses from all physicians, regardless of the control or intervention group. For the 2 surveys, we computed the score for each category by averaging all scores in each category. Then, we calculated the overall average score of the NASA-TLX and USE surveys for each physician. We excluded 1 physician from the survey data analysis who answered all questions as “NA” in the exit survey (N=33). We also analyzed audit log data for individual physicians and compared them with their survey results. We excluded 1 physician from the audit log data analysis whose postcoaching data were not available (N=33). All analyses were performed using SPSS, version 28.0 (SPSS Inc). Results were deemed statistically significant at *P*<.05 and marginally significant at *P*<.1.

## Results

A total of 34 pediatric physicians were enrolled and randomized. Of the 34 physicians, 15 (44%) were primary care pediatricians, 19 (56%) were women, 24 (70%) practiced at the medical center, 26 (76%) had 5-10 years of experience using Epic, and 17 (50%) reported using Epic for 20-40 hours a week. Groups AB and BA had 17 (50%) physicians each, with a similar subgroup distribution (Table 1).

**Table 1:** Study Participant Characteristics

Participants	All		Group AB		Group BA	
	N	%	N	%	N	%
Sex						
Male	15	44	8	47	7	41
Female	19	56	9	53	10	59
Role						
Assistant professor	13	38	6	35	7	41
Associate professor	6	18	3	18	3	18
Professor	5	15	2	12	3	18
Community pediatrics	10	29	6	35	4	24
Type						
Primary care pediatrician	15	44	8	47	7	41
Specialty pediatrician	19	56	9	53	10	59
Years of Epic experience, y						
0-5	7	21	4	24	3	18
5-10	26	76	12	71	14	82
>10	1	3	1	6	0	0
Estimated number of hours spent in Epic weekly, h						
0-20	8	24	4	24	4	24
21-40	17	50	7	41	10	59
41-60	7	21	4	24	3	18
>61	2	6	2	12	0	0
Total	34	100	17	50	17	50

### EHR Proficiency

During the initial intervention, the average (standard deviation [SD]) proficiency score of the interventional group (group AB) increased by 8.9% from 6.46 (1.34) to 6.83 (1.11) (pre-post difference, 0.37; 95% CI, −0.35 to 1.09; *P*=.381), whereas the average (SD) proficiency score of group BA (control) increased by 4.2% from 5.46 (1.93) to 5.56 (1.76) (pre-post difference, 0.11; 95% CI, −0.35 to 0.56; *P*=.691).

For the crossover intervention, the average (SD) proficiency score for group BA (intervention) significantly increased by 11.2% from 5.62 (1.73) to 5.94 (1.72) (pre-post difference, 0.31; 95% CI, −0.11 to 0.74; *P*=.05), whereas the average (SD) proficiency score for group AB (control) decreased by 0.43% from 6.94 (1.05) to 6.91 (1.12) (pre-post difference, −0.03; 95% CI, −0.23 to 0.17; *P*=.687; Figure).

**Figure:**
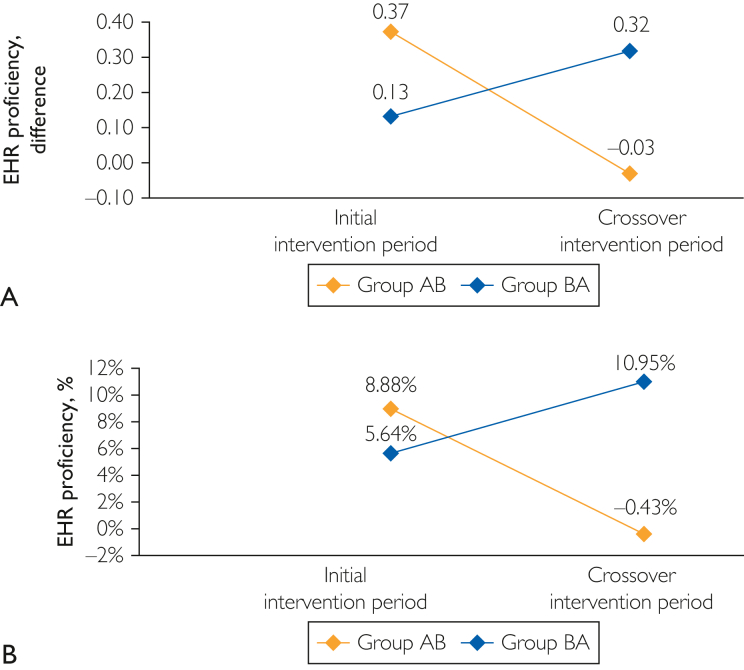
A, Difference in physicians’ proficiency score 3 months before and after the tailored coaching intervention for groups AB and BA during the initial and crossover intervention periods. B, Percent difference in physicians’ proficiency score 3 months before and after the tailored coaching intervention during the initial and crossover intervention periods.

The average (SD) Epic proficiency score of all the participating physicians significantly increased after the coaching intervention compared with that before the intervention (6.05 [1.5] vs 6.40 [1.6], respectively; *P*=.046). After the intervention, the average (SD) proficiency score significantly increased in the group of physicians who received coaching on time-based metrics (5.92 [1.7] vs 6.41 [1.6], *P*=.039). However, the average (SD) proficiency score remained the same before and after the intervention for physicians who received coaching to improve note length (6.36 [1.2] vs 6.37 [1.4], respectively; *P*=.575).

Male physicians showed consistent and significant improvements from the time they received the coaching intervention. The proficiency scores for the male physicians significantly improved after the intervention (5.58 [1.58] vs 6.24 [1.70]; *P*=.02; Table 2). Physicians with more than 5 years of experience with Epic showed significant improvement in proficiency scores after coaching (5.99 [1.68] vs 6.42 [1.56]; *P*=.038). Pediatric specialty physicians showed a significant increase in EHR proficiency after coaching (5.61 [1.51] vs 5.96 [1.51]; *P*=.043). Professors at the assistant professor rank had a significant increase in proficiency scores after coaching (5.65 [0.73] vs 6.32 [0.76]; *P*=.023).

**Table 2:** Subgroup analysis of Pre-Post Coaching Intervention Results on Electronic Health Record (EHR) Proficiency Score, Perceived EHR Workload, and Perceived EHR Usability^a^

Participants	EHR proficiency score				Group AB				Group BA			
	N (%)	Preintervention	Postintervention	*P* value	N (%)	Preintervention	Postintervention	*P* value	N (%)	Preintervention	Postintervention	*P* value
Sex												
Male	14 (42)	5.58 (1.58)	6.24 (1.70)	.026^b^	8 (47)	5.88 (1.08)	6.62 (1.3)	.40	6 (37)	4.83 (1.88)	5.28 (1.82)	.12
Female	19 (58)	6.49 (1.53)	6.61 (1.34)	.38	9 (53)	6.74 (1.34)	7.12 (0.86)	.68	10 (63)	6.09 (1.42)	6.32 (1.53)	.24
Type												
Primary care	15 (45)	6.69 (1.64)	7.03 (1.30)	.5	8 (47)	7.17 (1.45)	7.49 (0.88)	.89	7 (44)	6.22 (1.6)	6.54 (1.47)	.13
Specialty	18 (55)	5.61 (1.42)	5.96 (1.51)	.04^b^	9 (53)	5.82 (0.81)	6.24 (0.95)	.21	9 (56)	5.14 (1.66)	5.46 (1.75)	.21
Experience with Epic, y												
0-5	7 (21)	6.51 (1.29)	6.55 (1.33)	.61	4 (24)	6.86 (1.21)	6.96 (0.9)	.72	2 (12)	5.99 (1.5)	5.67 (1.85)	.66
>5	26 (79)	5.99 (1.68)	6.42 (1.56)	.04^b^	13 (76)	6.33 (1.35)	6.78 (1.16)	.46	14 (88)	5.56 (1.75)	5.97 (1.69)	.03^b^
Professional role												
Community pediatrics	10 (30)	7.19 (1.43)	7.51 (1.18)	.72	6 (35)	7.21 (1.35)	7.72 (0.81)	.75	4 (24)	7.16 (1.54)	7.19 (1.52)	.07^c^
Assistant professor	12 (36)	5.65 (0.73)	6.32 (0.76)	.02^b^	6 (35)	4.89 (0.06)	4.92 (0.03)	.35	6 (37)	5.59 (0.67)	6.26 (0.85)	.08^c^
Associate professor	6 (18)	6.18 (1.41)	5.95 (1.46)	.60	3 (18)	7.11 (1.37)	7.14 (0.75)	1	3 (18)	5.23 (0.57)	4.75 (0.9)	.29
Professor	5 (15)	4.92 (2.27)	5.26 (2.11)	.08^c^	2 (12)	5.89 (0.62)	6.41 (0.57)	.66	3 (18)	3.99 (2.34)	4.79 (2.33)	.11
Time spent using Epic (h/wk)												
0-20	7 (21)	6.00 (1.36)	6.12 (0.61)	.499	4 (24)	6.48 (1.4)	5.81 (0.53)	.068	3 (18)	5.47 (0.81)	6.31 (0.42)	.109
21-40	17 (52)	5.66 (1.73)	6.04 (1.78)	.113	7 (41)	6.4 (1.52)	7.1 (1.18)	.128	10 (64)	5.04 (1.64)	5.22 (1.68)	.386
>40	9 (27)	7.01 (1.14)	7.48 (0.86)	.260	6 (36)	6.5 (1.03)	7.19 (0.88)	.463	3 (18)	7.68 (0.88)	7.94 (0.54)	.109

a EHR, electronic health record.

b *P*<.05.

c *P*<.1.

### User Experience

#### EHR Workload

The average (SD) perceived EHR workload among the participants decreased after the coaching sessions compared with that before the sessions (50.89 [16.4] vs 46.66 [15.3], respectively; *P*=.06; Table 3). The average workload decreased in all the NASA-TLX survey variables, except for the “temporal demand” variable. The largest decrease was in the performance while using EHRs (44.24 vs 38.18; *P*=.349).

**Table 3:** Subgroup Analysis of Pre-Post Coaching Intervention Results on Electronic Health Record Workload (NASA Task Load Index) and Usability (Usability, Satisfaction, and Ease of Use) for All Participants, Group AB, and Group BA^a^^,^^b^^,^^c^

	All			Group AB			Group BA		
EHR tailored coaching intervention	Preintervention	Postintervention	*P* value	Preintervention	Postintervention	*P* value	Preintervention	Postintervention	*P* value
	(n=33)	(n=33)		(n=17)	(n=17)		(n=16)	(n=16)	
EHR workload (NASA-TLX)									
Mental demand	53.33 (22.98)	50.45 (19.24)	.34	49.12 (23.47)	50.58 (21.68)	.66	57.81 (21.57)	50.31 (16.24)	.16
Physical demand	49.24 (27.69)	43.03 (24.92)	.09	49.11(28.65)	46.47 (27.85)	.53	49.37 (26.62)	39.37 (20.75)	.09
Temporal demand	54.70 (23.58)	55.00 (23.26)	.94	57.94 (24.37)	60.29 (24.16)	.69	51.25 (22.18)	49.37 (20.83)	.65
Performance	44.24 (24.59)	38.18 (21.70)	.35	42.35 (24.38)	37.94 (24.37)	.94	46.25 (24.65)	38.43 (18.43)	.24
Effort	54.85 (19.21)	49.09 (19.90)	.14	50.29 (19.73)	47.94 (21.96)	.68	59.68 (17.36)	50.31 (17.36)	.11
Frustration	48.94 (26.19)	44.24 (23.94)	.22	48.82 (30.41)	49.7 (24.28)	.64	49.06 (20.78)	38.43 (22.13)	.14
Total average score	50.89 (16.22)	46.66 (15.16)	.06^d^	49.6 (18.5)	48.82 (16.75)	.72	52.24 (13.24)	44.37 (12.85)	.02^e^
EHR usability (USE)									
Usefulness	4.22 (1.20)	4.57 (1.19)	.007^e^	4.38 (1.08)	4.9 (1)	.006^e^	4.06 (1.29)	4.18 (1.24)	.26
Ease of use	3.89 (1.35)	4.16 (1.25)	.31	4.08 (1.35)	4.32 (1.25)	.65	3.66 (1.29)	3.96 (1.2)	.22
Ease of learning	4.56 (1.22)	4.69 (1.33)	.33	4.71 (1.23)	5.04 (1.19)	.04^e^	4.44 (1.18)	4.29 (1.34)	.46
Satisfaction	3.85 (1.42)	4.12 (1.44)	.02^e^	4.12 (1.28)	4.33 (1.45)	0.14	3.53 (1.55)	3.75 (1.26)	.06^e^
Total average score	4.10 (1.21)	4.34 (1.19)	.10^d^	4.27 (1.17)	4.57 (1.13)	0.03^e^	3.94 (1.22)	4.06 (1.19)	.92

a EHR, electronic health record; NASA-TLX, NASA Task Load Index; USE, Usability, Satisfaction, and Ease of Use.

b For NASA-TLX, a positive difference between preintervention and postintervention scores demonstrates improvement.

c For USE, a negative difference between preintervention and postintervention scores demonstrates improvement.

d *P*<.05.

e *P*<.1.

Twenty-one physicians (63.6%) reported that their workload had been reduced after the coaching session, whereas 12 physicians (36.4%) perceived their EHR workload to be higher after the intervention. For the physicians’ group that reported that their workload had been reduced, the average (SD) workload decreased from 54.38 (16.4) to 42.91 (13.2). The group that reported that their workload had increased after the coaching session had an average workload increase from 45.41 (16.8) to 52.44 (10.8).

#### EHR Usability

After the coaching intervention, the average (SD) score for perceived EHR usability improved compared with that before the intervention (4.10 [1.23] vs 4.34 [1.21], respectively; *P*=.1; Table 3). A total of 19 physicians (58%) reported that their satisfaction increased after the coaching session from an average of 3.90 (1.3) to 4.52 (1.2). The average score for each of the 4 USE subcategories increased after the coaching intervention. Among the 4 subcategories, EHR usefulness and EHR satisfaction significantly improved such that the average score (SD) for usefulness increased from 4.22 (1.20) to 4.57 (1.19) (*P*=.007) and that for satisfaction increased from 3.85 (1.42) to 4.12 (1.44) (*P*=.02) (Table 2).

### Subgroup Analysis

Similar to proficiency scores, both EHR workload and usability significantly improved after the coaching intervention among male physicians to the extent that the perceived EHR workload significantly decreased (50.78 [15.95] vs 45.16 [15.80]; *P*=.047) and the perceived EHR usability significantly increased (3.86 [1.22] vs 4.31 [1.14]; *P*=.023) (Table 4).

**Table 4:** Subgroup Analysis of Pre-Post Coaching Intervention Results on Perceived Electronic Health Record Workload (NASA Task Load Index) and Perceived Electronic Health Record Usability (Usability, Satisfaction, and Ease of Use)^a^

Participants	N (%)	Perceived EHR workload (NASA-TLX)			Perceived EHR usability (USE)		
		Preintervention	Postintervention	*P* value	Preintervention	Postintervention	*P* value
Sex							
Male	15 (45)	50.78 (15.95)	45.16 (15.80)	.047^b^	3.86 (1.22)	4.31 (1.14)	.02^b^
Female	18 (55)	50.98 (16.45)	47.91 (14.48)	.42	4.30 (1.16)	4.37 (1.23)	.91
Type							
Primary care	14 (42)	51.49 (16.51)	48.15 (15.55)	.33	4.58 (1.04)	4.72 (1.08)	.30
Specialty	19 (58)	50.44 (16.00)	45.57 (14.76)	.095^c^	3.74 (1.20)	4.06 (1.19)	.17
Experience with Epic, y							
0-5	6 (18)	42.63 (8.79)	39.44 (6.53)	.60	4.90 (0.76)	5.12 (0.83)	.46
>5	27 (82)	52.72 (16.91)	48.27 (16.04)	.07^c^	3.92 (1.22)	4.17 (1.19)	.16
Professional role							
Community pediatrics	9 (27)	48.15 (17.21)	46.20 (15.71)	.26	4.96 (0.75)	5.11 (0.67)	.52
Assistant professor	13 (39)	48.14 (15.07)	44.42 (17.10)	.22	4.05 (1.13)	4.44 (1.29)	.09^c^
Associate professor	6 (18)	60.14 (18.56)	53.75 (10.97)	.35	3.04 (0.93)	3.20 (0.65)	.75
Professor	5 (15)	51.53 (7.98)	44.83 (9.52)	.14	3.97 (1.25)	4.08 (1.00)	.89
Time spent using Epic (h/wk)							
0-20	8 (24)	51.25 (12.03)	48.75 (16.47)	.67	4.08 (1.03)	4.34 (1.18)	.48
21-40	16 (48)	51.25 (17.59)	43.02 (12.65)	.02^b^	4.03 (1.40)	4.33 (1.25)	.13
>40	9 (27)	49.91 (16.89)	51.30 (16.37)	.95	4.25 (0.95)	4.37 (1.09)	.59

a EHR, electronic health record; NASA-TLX, NASA Task Load Index; USE, Usability, Satisfaction, and Ease of Use.

b *P*-value indicate statistical significance.

c *P*-value indicate marginal significance.

The intervention significantly increased the EHR workload for physicians who spent 21-40 hours using Epic (51.25 [17.59] vs 43.02 [12.65]; *P*=.02). After coaching, the EHR workload improved for pediatric specialists (50.44 [16] vs 45.57 [14.76]; *P*=.095) and pediatricians with more than 5 years of experience with Epic (52.72 [16.91] vs 48.27 [16.04]; *P*=.073). Assistant professors’ EHR usability rating improved after coaching (4.05[1.13] vs 4.44 [1.29]; *P*=.087).

## Discussion

To our knowledge, this 12-month, randomized, crossover trial is the first to use EHR audit log data to inform the development of a tailored EHR coaching intervention to improve physicians’ proficiency and users’ experience in EHRs. Tailored EHR coaching improved both the primary outcomes, with more significant improvements in the crossover phase. During the initial intervention, the intervention group had substantially improved EHR proficiency scores compared with the control group. During the crossover period, the intervention group had significantly improved EHR proficiency scores compared with control group, which had almost no change.

When both the intervention groups were combined, we found that the average EHR proficiency scores significantly improved among the physicians after they received tailored EHR coaching. In particular, we found that the EHR proficiency levels significantly improved among physicians who received coaching to improve time-based metrics. We report that the subgroups that demonstrated significant improvements in EHR proficiency after coaching were physicians who were men, specialty pediatricians, had more than 5 years of experience using Epic, assistant professors, and full professors. Similarly, both perceived EHR workload and usability improved after coaching.

We found a substantial improvement in the perceived EHR workload after the coaching intervention, highlighting that physician performance in EHRs was the largest area of improvement. Nearly half of the physicians who reported a decrease in the perceived workload also demonstrated objective improvement in their audit log data after coaching. This improvement in both subjective and objective parameters was the largest in the group focused on EHR documentation length compared with time-based measures. The discrepancy between subjective and objective results may have been due to differing expectations by the physicians from the intervention. Additionally, objective changes between before and after the intervention may need to be larger to become noticeable for physicians.

Additionally, we found a considerable improvement in the perceived EHR usability among the physicians after coaching. Both the usefulness of and satisfaction with EHRs significantly improved after the intervention. These findings suggest that tailored coaching facilitates a better understanding of available tools in EHRs and reduces frustration. Previous studies have found comparable improvement in EHR satisfaction with previous one-on-one EHR and department-focused coaching using subjective measures, which demonstrated improved satisfaction, knowledge, and comfort with EHRs.^6^^,^^17^^,^^38^

In general, we found that the physicians became more familiar with efficiency tools available in EHRs after the tailored coaching intervention, which validates previous calls to tailor EHR coaching for physicians’ needs. This adds to the existing knowledge that EHR training, in general, improves physician proficiency.^39^^,^^40^ Approximately 85%-98% of physicians in a self-reported study showed improved efficiency, time savings, and in-basket workload after a 3-day, intense, classroom-based EHR training.^11^ Another study demonstrated statistically significant improvement in self-reported EHR efficiency and workload based on presurveys and postssurveys.^17^ This may explain the decrease in perceived workload and improved usability in the physicians after the coaching session.

We report that men experienced greater improvement from the time of the coaching session than women. Previous literature reported no significant differences in EHR usage based on the sex of providers; however, women were reported to have a higher likelihood of burnout than men.^27^ It is plausible that other burnout-related factors may have impeded the effect of the coaching session on women. Because this study relied on a single male coach to provide the sessions, more studies are needed to examine whether the sex of the coach had an impact on outcomes.

All coaching sessions were conducted virtually. The initial plan was to deliver peer-to-peer coaching during elbow support. However, because of the pandemic, we needed to change to virtual sessions. The benefits of virtual coaching included the ability to record the sessions with screen capture, extra flexibility to accommodate providers’ schedules, and removal of transportation logistics. The limitations of virtual coaching included the inability to share 2 screens at the same time to provide support to providers’ as they customized their screens, lack of face-to-face interaction, and dependency on technology such as computers and internet bandwidth. Future studies should explore whether there are differences in outcomes between in-person and virtual coaching.

### Limitations

This study has several limitations. Our study was conducted at an academic medical institution using 1 EHR system (Epic). The EHR coaching session was conducted by 1 Epic-certified builder. Therefore, the study may not necessarily be representative of other medical institutions or EHR systems. Additionally, although audit log data were used for objective measures, the study did not independently verify the accuracy of audit log data. Although there was a washout period of 6 months of difference between the initial and crossover interventions, the acquisition of audit logs overlapped in 1 month of the study (ie, February 2021), which may have had an effect on the data outcomes of this month. Some physicians had missing data from February 2021, and researchers were unable to obtain replaceable data from the data contributor. The study also did not account for any potential vacation, leave of absence, parental leave, or days off that the physicians may have had during the study, although any missing values were removed when data analysis was performed.

## Conclusion

To our knowledge, this is the first study to use EHR audit log data to objectively develop a tailored coaching intervention. Our study found that tailored EHR coaching improved EHR proficiency, reduced perceived workload, and improved EHR usability among pediatric physicians. The findings of this study are relevant to decision makers and policymakers interested in provider well-being by optimizing EHR use through effective EHR training strategies.

## Potential Competing Interests

The authors report no competing interests.
